# Inferior Vena Cava Filter Retrieval Rates Associated With Passive and Active Surveillance Strategies Adopted by Implanting Physicians

**DOI:** 10.1001/jamanetworkopen.2023.3211

**Published:** 2023-03-16

**Authors:** Emily Sterbis, Jonathan Lindquist, Alexandria Jensen, Michael Hong, Shane Gupta, Robert Ryu, P. Michael Ho, Premal Trivedi

**Affiliations:** 1 Department of Radiology, University of Colorado Anschutz Medical Campus, Aurora; 2 Department of Biostatistics and Informatics, Colorado School of Public Health, Aurora; 3 Department of Radiology, University of Southern California Keck School of Medicine, Los Angeles; 4 Division of Cardiology, VA Eastern Colorado Health System, Aurora

## Abstract

**Importance:**

Inferior vena cava filters are commonly implanted and infrequently retrieved. Nonretrieval contributes to significant morbidity, motivating US Food and Drug Administration and multisociety communications emphasizing the need for improved device surveillance. Current guidelines suggest that implanting physicians and referring physicians should be responsible for device follow-up, but it is not known whether shared responsibility contributes to lower retrieval.

**Objective:**

To determine if primary responsibility for follow-up care assumed by the implanting physician team is associated with increased device retrieval.

**Design, Setting, and Participants:**

This retrospective cohort study examined a prospectively collected registry of patients with inferior vena cava filters implanted from June 2011 to September 2019. Medical record review and data analysis was completed in 2021. The study included 699 patients who underwent implantation of retrievable inferior vena cava filters at an academic quaternary care center.

**Exposures:**

Prior to 2016, implanting physicians had a passive surveillance strategy whereby letters highlighting indications for and the need for timely retrieval were mailed to patients and ordering clinicians. Starting in 2016, implanting physicians assumed active responsibility for surveillance, whereby candidacy for device retrieval was assessed periodically via phone calls and retrieval scheduled when appropriate.

**Main Outcomes and Measures:**

The main outcome was the odds of inferior vena cava filter nonretrieval. Within regression modeling of the association between the surveillance method and nonretrieval, additional covariates of patient demographics, concomitant malignant neoplasm, and presence of thromboembolic disease were included.

**Results:**

Of the 699 patients who received retrievable filter implants, 386 (55.2%) were followed up with passive surveillance, 313 (44.8%) with active surveillance, 346 (49.5%) were female, 100 (14.3%) were Black individuals, and 502 (71.8%) were White individuals. The mean (SD) age at filter implantation was 57.1 (16.0) years. Mean (SD) yearly filter retrieval increased following the adoption of active surveillance, from 190 of 386 (48.7%) to 192 of 313 (61.3%) (*P* < .001). Fewer filters were deemed permanent in the active group vs passive group (5 of 313 [1.6%] vs 47 of 386 [12.2%]; *P* < .001). Age at the time of implantation (OR, 1.02; 95% CI, 1.01-1.03), concomitant malignant neoplasm (OR, 2.18; 95% CI, 1.47-3.24), and passive contact method (OR, 1.70; 95% CI, 1.18-2.47) were associated with increased odds of filter nonretrieval.

**Conclusions and Relevance:**

The findings of this cohort study suggest that active surveillance by implanting physicians is associated with improved inferior vena cava filter retrieval. These findings support encouraging physicians who implant the filter to take primary responsibility for tracking and retrieval.

## Key Points

**Question:**

Does active surveillance by implanting physicians improve retrieval of inferior vena cava filters?

**Findings:**

In this cohort study of 699 patients, 2 methods of structured follow up were compared after retrievable filter implantation: active (candidacy for device retrieval assessed periodically via phone calls and retrieval scheduled when appropriate) vs passive (certified letters highlighting indications for and need for timely retrieval mailed to patients and ordering clinicians). Filter retrieval improved significantly from 48.4% to 61.6% with adoption of active surveillance by implanting physicians.

**Meaning:**

These findings suggest that active surveillance, whereby implanting physicians take primary responsibility for filter follow up, was associated with significantly improved device retrieval.

## Teaser

This cohort study assesses whether active surveillance follow-up by physicians who implant retrievable inferior vena cava filters is associated with increased rates of device retrieval.

## Introduction

Retrievable inferior vena cava (IVC) filters are designed to prevent venous thromboembolism-related morbidity and mortality when anticoagulation is contraindicated. IVC filters are frequently implanted but rarely retrieved, with national estimates of 12% to 18% aggregate retrieval.^1,2^ IVC filters are clinically most beneficial if retrieved within 90 days.^1,3^ Delayed retrieval and nonretrieval can lead to avoidable complications, including filter migration (up to 11.8%), fracture (up to 21%), recurrent deep venous thrombus (1.9%-14.5%), pulmonary embolism (0.5%-12%), and vessel or organ perforation (up to 12.4%).^3^ Thus, 2010 and 2014 FDA advisories and recent multisociety guidelines recommend that implanting physicians and referring physicians be responsible for patient follow-up and timely IVC filter retrieval to reduce complications.^4^ Prior single-institution studies have corroborated the importance of device surveillance, reporting improved filter retrieval with any form of follow up. This includes surveillance strategies involving both passive and active involvement of the implanting physician team. Tested passive surveillance methods include routine medical record review with letters sent to patients who qualify for IVC filter retrieval,^5^ reminders that filters should be retrieved promptly in the IVC filter implantation report along with a reminder to the referring clinician,^6^ and prescheduling filter retrieval at the time of implantation.^7^ Active surveillance methods shown to increase filter retrieval include the dedication of multidisciplinary tracking teams with a routine clinic or phone follow up^8^ and the institution of a clinic to facilitate periodic assessment for device retrieval.^9,10^

Federal communications imply shared responsibility between implanting physicians and referring clinicians for device surveillance. We hypothesized that active surveillance, in which primary responsibility for device follow up and retrieval is taken on by the implanting team, would result in improved retrieval rates compared to passive surveillance.

## Methods

This cohort study followed the Strengthening the Reporting of Observational Studies in Epidemiology (STROBE) reporting guideline. This study was deemed exempt from review by the institutional review board at the University of Colorado, and informed consent was waived because patient data were deidentified prior to dissemination. Data were retrospectively analyzed from our prospectively collected single-institution IVC filter registry tracking patients with filters implanted from June 2011 to September 2019. All patients with a filter implanted during this time were included in the study.

All patients with an IVC filter implantation were added to an institutional database for follow-up by the interventional radiology department. The registry was built and maintained by a group of interventional radiology advanced practice clinicians. Patients were enrolled at the time of IVC filter implantation through submission of a form by the implanting physician, containing the following information: date of implantation, implanting physician name and contact information, patient medical record number, reason for device implantation, referring physician name and contact information, and space for free-text comments. To check for any missing documentation, implantation procedures were independently retrieved, approximately monthly, from the institutional billing group and cross-checked against entered registry data. Additional relevant information was then added by the reviewing advanced practice clinician, including type of filter (retrievable, permanent, or convertible), resumption of anticoagulation (yes or no), dose and type of anticoagulant, whether the filter has been retrieved, and free text notes on any communication with the referring clinician or patient. In these notes, patients and/or family members who could not be contacted despite at least 2 attempts were deemed lost to follow-up.

This quality improvement database collected as part of routine clinical care was used to identify all patients during the study period. Medical record review was performed to obtain additional patient factors, including date of birth (to determine age at the time of filter implantation), sex, race and ethnicity entered through patient registration, primary insurance, residence zip code, and distance from patient home to the hospital. Patient race and ethnicity are captured in the electronic medical record at time of initial registration and are typically self-reported. They were extracted to examine any associated differences in device retrieval. Patient races, including American Indian or Alaska Native, Native Hawaiian or Pacific Islander, or more than 1 race, are included in the “other” group.

Additional focused medical record review was performed during the retrospective review to ensure the accuracy of collected data for research purposes. Indication for filter implantation was examined again in notes from the days preceding implantation. We noted the presence or absence of thromboembolic disease (deep vein thrombosis and/or pulmonary embolism), how the diagnosis was made, the date of diagnosis, imaging study type, and findings supporting it. The presence of contraindication to anticoagulation was tracked. Key associated diagnoses of concomitant malignant neoplasm, admission following major trauma, or prior to major surgery were also extracted. The type of IVC filter implanted was cross-checked against implantation procedural notes and included brand name and whether the filter was retrievable, permanent, or convertible. Whether the IVC filter was retrieved and the date of retrieval were primarily extracted from the registry. Data were cross-checked for accuracy against the electronic medical record via the presence of a retrieval procedure or documentation of retrieval at another institution. When relevant, reasons for nonretrieval were noted in the IVC filter registry and cross-checked against the electronic medical record. If filters were retrieved, the location of filter retrieval, and the retrieval date to calculate filter dwell time (time from implantation to retrieval) were extracted from the medical record. Patient date of death was primarily extracted from institutional medical records. It was supplemented with the social security death index to minimize missing data.

Implantation characteristics, retrieval status, and predictors of nonretrieval were examined for 2 types of structured follow-up: passive (June 2011 to January 2016) and active (February 2016 to September 2019) management by the implanting physician team. The historic passive surveillance involved periodic review of the IVC filter registry, mailing patients a certified letter (eAppendix in Supplement 1), and sending the referring clinicians a message on the importance of and indications for IVC filter retrieval. The decision for retrieval required active decision-making from the referring physicians or clinicians primarily responsible for patient care. Active surveillance involved structured follow up with approximately monthly communication from the implanting physician team to the patient and referring or primary care clinicians eliciting periodic updates on clinical status, including criteria for retrieval. Decision for device retrieval could be made solely by the implanting physician team when clinical criteria were met (typically resolution of thrombus or resumption of anticoagulation), whether that information came from the patient or other members of their care team. Active surveillance focused on more communication with clinicians without sending letters to patients and rarely calling patients, typically only if clinicians in the system no longer had consistent contact. There was no specific timeline for each step in follow-up for both periods of surveillance. Rather, an advanced practice clinician from the implanting physician team reviewed active patients with implanted filters in the registry once a month.

### Statistical Analysis

A *t* test was used for comparison of continuous variables, and a Fisher exact test was used for categorical variables, comparing demographic and filter-related characteristics between the passive and active groups in the Table. An initial assessment of aggregated filter retrieval proportion by study year was conducted, with a 2-sided *t* test performed to compare retrieval rates in the passive and active surveillance eras. This was followed by a logistic regression fit to test the association between the surveillance method and filter nonretrieval, with additional covariates of patient age at implantation (mean-centered), patient sex, race, primary insurance, distance to the implanting hospital, concomitant malignant neoplasm, and presence of thromboembolic disease included as potential confounders. All model variables were chosen a priori based on clinical relevance, not based on statistical significance. Statistical significance was set to the standard of *P* < .05. All analyses were conducted in R version 4.2.1 (R Project for Statistical Computing).

**Table.** Descriptive Statistics—Patient Demographics, Indications, and IVC Filter Types

Characteristic	No. (%)			*P* value
	Overall (n = 699)	Active (n = 313)	Passive (n = 386)	
Patient age at implantation, mean (SD), y	57.1 (16.0)	56.4 (16.2)	57.8 (15.8)	.26
Patient sex				
Female	346 (49.5)	153 (48.9)	193 (50.0)	.82
Male	353 (50.5)	160 (51.1)	193 (50.0)	
Patient race and ethnicity				
African American or Black,				.06
Not Hispanic	99 (14.2)	53 (16.9)	46 (11.9)	
Hispanic	1 (0.1)	1 (0.3)	0	
Asian				
Not Hispanic	10 (1.4)	1 (0.3)	9 (2.3)	
Hispanic	0	0	0	
Hispanic	60 (8.6)	28 (8.9)	32 (8.3)	
White				
Not Hispanic	500 (71.5)	219 (69.9)	281 (72.8)	
Hispanic	2 (0.3)	0	2 (0.5)	
Other race^a^				
Not Hispanic	13 (1.9)	6 (1.9)	7 (1.8)	
Hispanic	2 (0.3)	0	2 (0.5)	
Missing	12 (1.7)	5 (1.6)	7 (1.8)	
Primary insurance				
Medicare or Medicaid	369 (52.8)	179 (57.2)	190 (49.2)	.03
Private	226 (32.3)	101 (32.3)	125 (32.4)	
Tricare	30 (4.3)	12 (3.8)	18 (4.7)	
None	71 (10.2)	21 (6.7)	50 (13.0)	
Missing	3 (0.4)	0	3 (0.8)	
Distance to implanting hospital, mean (SD), miles	117.3 (243.4)	139.5 (283.3)	99.3 (204.0)	.06
Thromboembolic disease				
None	51 (7.3)	15 (4.8)	36 (9.3)	.07
DVT	306 (43.8)	139 (44.4)	167 (43.3)	
Pulmonary embolism with or without DVT	341 (48.8)	158 (50.5)	183 (47.4)	
Missing	1 (0.1)	1 (0.3)	0	
Indication				
VTE with contraindication/failed AC	374 (53.5)	184 (58.8)	190 (49.2)	<.001
VTE and surgery planned	116 (16.6)	72 (23.0)	44 (11.4)	
VTE and low pulmonary reserve	85 (12.1)	18 (5.8)	67 (17.4)	
No VTE and trauma	11 (1.6)	10 (3.2)	1 (0.3)	
No VTE and high risk for surgery/bleed	37 (5.3)	5 (1.6)	32 (8.3)	
IVC/high risk thrombus	21 (3.0)	2 (0.6)	19 (4.9)	
Other, thrombolysis	45 (6.4)	22 (7.0)	23 (6.0)	
Missing	10 (1.4)	0	10 (2.6)	
Concomitant malignant neoplasm				
Yes	231 (33.0)	97 (31.0)	134 (34.7)	.42
No	457 (65.4)	208 (66.5)	249 (64.5)	
Missing	11 (1.6)	8 (2.6)	3 (0.8)	
Retrievable filter type				
ALN	96 (13.7)	80 (25.6)	16 (4.1)	<.001
Crux	8 (1.1)	1 (0.3)	7 (1.8)	
Gunther tulip	401 (57.4)	47 (15.0)	354 (91.7)	
Option	194 (27.7)	185 (59.1)	9 (2.3)	

Abbreviations: DVT, deep vein thrombosis; IVC, inferior vena cava; VTE, venous thromboembolism.

^a^ Patient races including American Indian or Alaska Native, Native Hawaiian or Pacific Islander, or more than 1 race are aggregated into the “other” group.

## Results

A total of 786 patients had inferior vena cava filters implanted during the study period, 699 of which were retrievable. The remaining patients with permanent or convertible filters were excluded since they were not intended for retrieval and were not followed by our surveillance program. There were 313 patients (44.8%) in the active surveillance group and 386 patients (55.2%) in the passive surveillance group. The mean (SD) patient age of the overall cohort was 57.1 years (16.0), and it did not differ significantly by surveillance type (Table). Patient sex also did not differ significantly by surveillance cohort; 346 of the 699 (49.5%) overall were female. A slightly greater proportion of non-Hispanic African American or Black patients were seen in the active group (53 of 313 [16.9%]) compared to the passive group (46 of 386 [11.9%]). However, the overall difference in race and ethnicity composition between the groups did not reach statistical significance.

While the mean (SD) distance from patient residence to the hospital was greater for the active group, the difference was not statistically significant (active: 139.5 [283.3] miles vs passive: 99.3 [204.0] miles; *P* = .06). More patients in the active surveillance group had filters implanted for the absolute indication of venous thromboembolism and inability to anticoagulate (active: 184 of 313 [58.8%] vs passive: 190 of 386 [49.2%]; *P* = .04). In terms of clinical indication, frequency of concomitant malignant neoplasm did not differ significantly between groups (active: 97 of 313 [31.0%] vs passive: 134 of 386 [34.7%]; *P* = .42). Patients in the passive group were more likely to have Gunther Tulip filters (active: 47/313 [15.0%] vs passive: 354/386 [91.7%]; *P* < .001) due to changing practice preferences over time.

**Figure 1.**
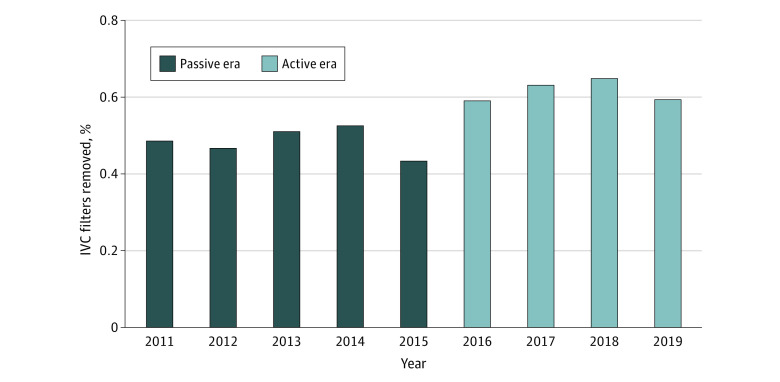
Bar Graph Demonstrating Percentage of IVC Filters Retrieved in Each Year of the Study IVC indicates inferior vena cava.

**Figure 2.**
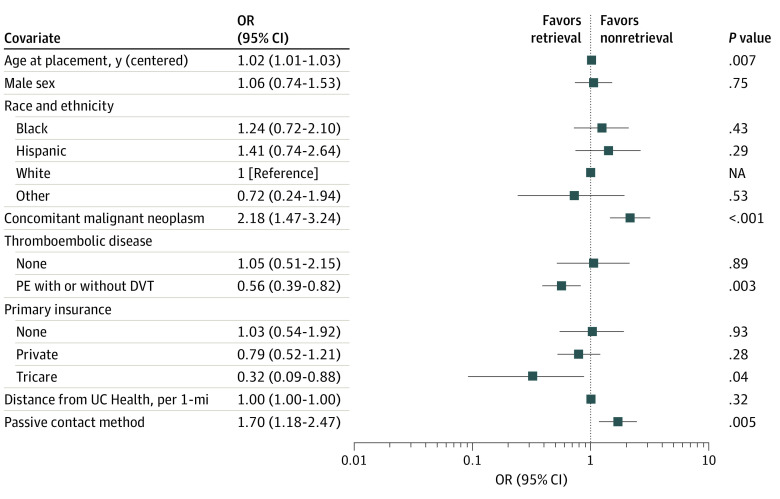
Forest Plot Demonstrating Logistic Regression of Variables for Nonretrieval DVT indicates deep vein thrombosis; OR, odds ratio; PE, pulmonary embolism; UC Health, University of Colorado Health.

Aggregate filter retrieval per year increased from 190 of 386 (48.7%) to 192 of 313 (61.3%) following the adoption of active surveillance (*P* < .001) (Figure 1). Importantly, retrieval rates increased immediately without any noticeable lag period, and the increase was sustained throughout the study period. Retrieval within 1 year from filter implantation was 386 (46.4%) in the passive group and 313 (58.1%) in the active group (*P* = .003). This difference was primarily driven by the decision to stop follow-up and deem the filter permanent in the passive group (active: 5 of 313 (1.6%) vs passive:47 of 386 [12.2%]; *P* < .001). A greater proportion of patients were lost to follow-up in the active group (active: 46 of 313 [14.7%] vs passive: 35 of 386 [9.1%]; *P* = .03). More patients died within 90 days of implantation in the passive group (active: 33 of 313 [10.5%] vs passive: 67 of 386 [17.4%]; *P* = .01). Median (Q1-Q3) dwell time was similar between groups (active: 82 [46.75-151.00] days vs passive: 55 [33.50-110.00] days; *P* = .77). Passive surveillance was associated with higher unadjusted odds of filter nonretrieval (odds ratio [OR], 1.72; 95% CI, 1.27-2.33; *P* < .001) in univariate analysis. Excluding patients who died within 90 days of filter implantation, multivariate logistic factors associated with nonretrieval (Figure 2) were age at the time of implantation (OR, 1.02; 95% CI, 1.01-1.03; *P* = .007), concomitant malignancy (OR, 2.18; 95% CI, 1.47-3.24; *P* < .001), and passive surveillance method (OR, 1.70; 95% CI, 1.18-2.47; *P* = .005). Conversely, an underlying indication of thromboembolic disease (OR, 0.56; 95% CI, 0.39-0.82; *P* = .003) and a primary insurer of Tricare (OR = 0.32; 95% CI. 0.09-0.88; *P* = .04) were associated with decreased odds of nonretrieval and, thus, increased odds of retrieval.

## Discussion

This cohort study examined the association between 2 methods of surveillance following IVC filter implantation and the associated odds of retrieval at a single quaternary care institution. While prior studies have demonstrated that any form of structured surveillance significantly improves IVC filter retrieval, this study compares 2 methods of surveillance against each other. A significant increase in yearly device retrieval proportion was observed with the implanting physician team taking primary responsibility for device surveillance, communication, and retrieval as compared with a passive surveillance strategy whereby the implanting primary served to facilitate information exchange and encouraged retrieval. In our experience, the ordering clinician often does not have continuity of care beyond the patient’s initial implantation encounter, which presents a significant hurdle in making an informed decision to retrieve the device, especially as the patient’s clinical status evolves between encounters. Active surveillance did not reduce the number of patients who were lost to follow-up but rather significantly reduced the number of patients allocated to permanent filtration. Multidisciplinary responsibility in the passive surveillance era was more resource-demanding since it required engagement from both implanting and referring physicians before a device could be retrieved. The challenge of making an informed decision for IVC filter retrieval involving referring physicians who may not know the patient’s evolving course after implantation may have resulted in fewer filters being retrieved and more being deemed permanent.

Prior studies have demonstrated improved retrieval rates with the institution of a dedicated filter follow-up clinic, which assumes an active surveillance burden on the implanting physician team.^8,9,10^ While previous studies have focused on the initial implementation of a single filter retrieval follow-up process,^5,7,8,9^ this study compares 2 different follow-up methods at the same institution. The more active surveillance method places greater responsibility on the implanting team to both follow patients with filters and enables them to make clinical decisions regarding whether or not the filter should be retrieved. Indications for filter retrieval include resolution of VTE risk or resumption of anticoagulation, both of which can be assessed effectively by implanting physicians in follow-up, as long as the necessary information is collected from the patient, medical record documentation, or other treating physicians. These findings support having the implanting physician team take primary responsibility for device follow up and retrieval.

### Limitations

Although this study represents a large sample size, its retrospective nature and single institution source are limitations to generalizability. Reasons for nonretrieval were assigned retrospectively through medical record review, thus limiting information to the categories reported here. Finally, the appropriateness of retrieval is generally left to clinical discretion and cannot be adjudicated with data analyzed retrospectively.

## Conclusions

These findings suggest that active surveillance, whereby primary responsibility for device surveillance and retrieval is taken on by the implanting physician team is associated with higher filter retrieval compared with passive surveillance involving shared responsibility between implanting and referring physicians. This study supports having the implanting physician team take primary responsibility for IVC filter tracking and retrieval.

## Notes

Supplement 1. **eAppendix.** Letter

Supplement 2.
Data Sharing Statement
