# Supplementary material for: Inferior Vena Cava Filter Retrieval Rates Associated With Passive and Active Surveillance Strategies Adopted by Implanting Physicians
Source: JAMA Netw Open. 2023 Mar 16;6(3):e233211. doi: 10.1001/jamanetworkopen.2023.3211 (PMC10020881; doi:10.1001/jamanetworkopen.2023.3211)
Supplement: Supplement 1. — eAppendix. Letter [file jamanetwopen-e233211-s001.pdf]

Supplemental Online Content

Sterbis E, Lindquist J, Jensen A, et al. Inferior vena cava filter retrieval rates associated with passive and active surveillance strategies adopted by implanting physicians. *JAMA Netw Open*. 2023;6(3):e233211. doi:10.1001/jamanetworkopen.2023.3211

eAppendix. Letter

This supplemental material has been provided by the authors to give readers additional information about their work.

© 2023 Sterbis E et al. *JAMA Network Open.*

eAppendix. Letter

On ***, you received an IVC (inferior vena cava) filter. It is part of your treatment for ***. The filter was placed in the large vein that returns blood to your heart from your legs. It traps blood clots and stops them from going to your lungs.

Your filter is "optionally retrievable." It can be removed at any time. It can also stay in place as long as you need it. Your medical providers will consider several factors in your case. They will work with you to decide whether your filter should stay in place or be removed.

The risks of leaving the filter in place are small, but you need to know about them. The filter can break. The filter, or pieces of the filter, can move. A clot can form around the filter. In rare cases, medical providers are unable to remove the filter.

The longer the filter is in place, the greater the risks become. That’s why you need to follow up with your medical providers, especially your primary care physician. Talk about the status of your filter. Ask whether it should be removed. That conversation will benefit both you and your medical providers.

If you have questions, please call me at *** or email ***. I am always happy to provide more information. I may also call you to check on the status of your filter.

© 2023 Sterbis E et al. *JAMA Network Open.*
