# Supplementary material for: Inferior Vena Cava Filter Retrieval Rates Associated With Passive and Active Surveillance Strategies Adopted by Implanting Physicians
Source: JAMA Netw Open. 2023 Mar 16;6(3):e233211. doi: 10.1001/jamanetworkopen.2023.3211 (PMC10020881; doi:10.1001/jamanetworkopen.2023.3211)
Supplement: Supplement 2. — Data Sharing Statement [file jamanetwopen-e233211-s002.pdf]

Data Sharing Statement

Sterbis. Inferior Vena Cava Filter Retrieval Rates Associated With Passive and Active Surveillance Strategies Adopted by Implanting Physicians. *JAMA Netw Open*. Published March 16, 2023. doi:10.1001/jamanetworkopen.2023.3211

Data
Data available: No
